# Extracorporeal Membrane Oxygenation Support for Cardiac Dysfunction Due to Kawasaki Disease Shock Syndrome

**DOI:** 10.3389/fped.2019.00221

**Published:** 2019-06-06

**Authors:** Han Zhang, Lijian Xie, Tingting Xiao

**Affiliations:** Department of Cardiology, Shanghai Children's Hospital, Shanghai Jiaotong University, Shanghai, China

**Keywords:** Kawasaki disease, Kawasaki disease shock syndrome, extracorporeal membrane oxygenation, cardiogenic shock, intravenous γ-globulin resistance

## Abstract

**Background:** Kawasaki disease (KD) is usually characterized as an inflammatory vasculitis during early childhood, which predominantly involves medium-sized arteries and is treated with intravenous γ-globulin (IVIG) and oral aspirin. KD with hemodynamic instability, characterized by systolic blood pressure decreasing by more than 20% below the normal range, is defined as Kawasaki disease shock syndrome (KDSS). The pathogenesis of KDSS is still not comprehensively understood. Life-threatening cardiogenic shock can occur during the acute phase of KDSS, while the mechanism of cardiac dysfunction due to KDSS is still controversial, and such cases are rarely reported. Here, we present the application of veno-arterial (VA) extracorporeal membrane oxygenation (ECMO) for cardiac function support of a child with KDSS. By doing so, it will be a reminder that KDSS can cause severe cardiac dysfunction, and we should stay vigilant at the early stage of the disease to distinguish KDSS from toxic septic shock in the first place and initiate the appropriate treatment at the right moment, in order to prevent such patients from having irreversible outcomes.

## Introduction

Kawasaki disease (KD) is an inflammation related to vasculitis and mostly involving medium-sized arteries (1). The incidence of Kawasaki disease shock syndrome (KDSS) among patients with KD ranges from 1.9 to 7.0 (2–5). Children with KDSS require hemodynamic support and intensive medical care (6). The diagnosis of KDSS can be easily ignored, which can sometimes lead to irreversible consequences (7). In industrialized countries, KD is the leading cause of acquired heart disease during early childhood, and may result in long-term, potentially severe cardiovascular sequelae (8). Patients who clinically present with high degree of fever, rash, conjunctivitis, and severe cardiac and other organ dysfunction, mimicking toxic septic shock syndrome, lead to a diagnosis of KDSS (9). Use of extracorporeal life support for cardiac failure should be considered for patients with evidence of inadequate end organ perfusion and oxygen delivery resulting from inadequate systemic cardiac output: (a) Hypotension despite maximum doses of two inotropic or vasopressor medications. (b) Low cardiac output with evidence of end organ mal-perfusion despite medical support as described above: persistent oliguria, diminished peripheral pulses. (c) Low cardiac output with mixed venous or superior caval central venous (for single ventricle patients) oxygen saturation < 50% despite maximal medical support. (d) Low cardiac output with persistent lactate >4.0 and persistent upward trend despite optimization of volume status and maximal medical management (10). The case we reported here describes the successful deployment of veno-arterial (VA) extracorporeal membrane oxygenation (ECMO) under such circumstance.

## Case Presentation

**Figure 1:**
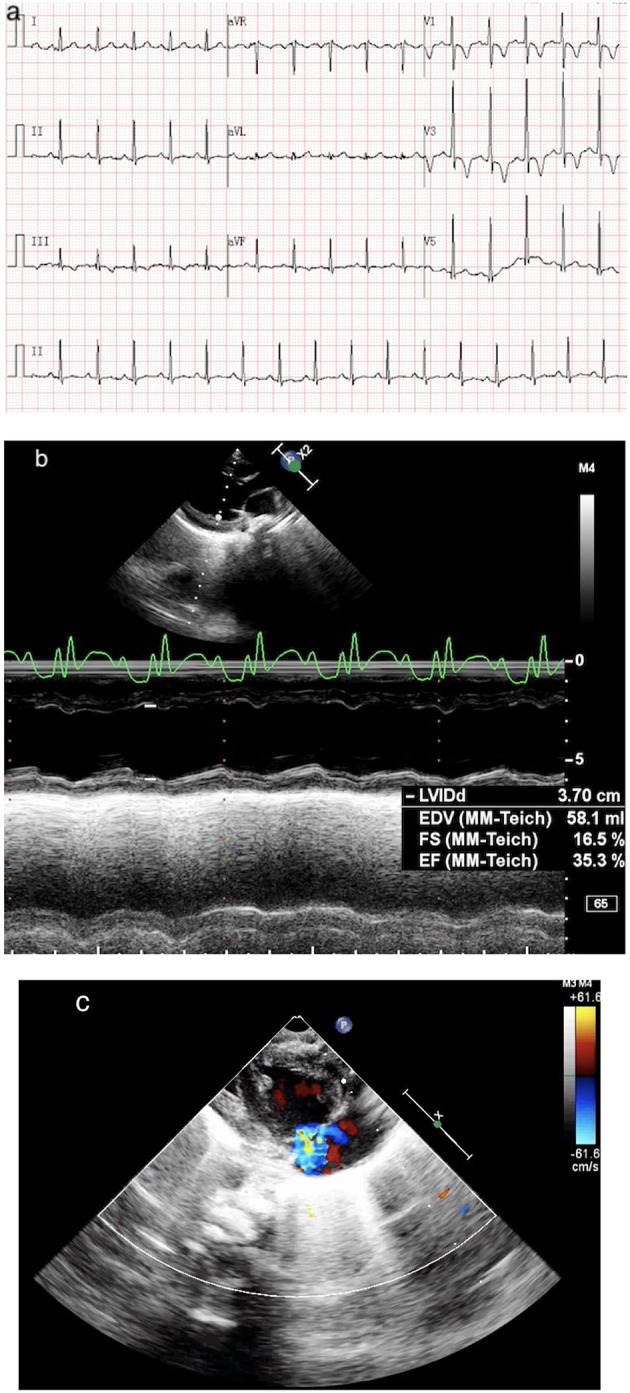
**(a)** ECG shows sinus tachycardia with lowering and inverted T wave on leads II, III, and avF. **(b)** Echo shows a dilated, poorly functioning left ventricle (EF 35%). **(c)** Echo shows a severe TR.

A 4-year-old girl (weight, 18 kg) with no medical history presented with 3 days of fever, 2 days of rash, and conjunctivitis. Physical examination revealed bilateral cervical lymphadenopathy and swelling of limb extremities. Chest and cardiac examination results were unremarkable. Laboratory test showed that the white blood cell (WBC) count was 12.50 × 10^9^/L, neutrophils ratio (NE%) was 70.8%, platelet count (PLT) was 121 × 10^9^/L, and C-reactive protein (CRP) was 127 mg/L. Erythrocyte sedimentation rate (ESR) was 90 mm. Serum albumin (ALB) and sodium were 38.17 g/L and 129 mmol/L, respectively. Troponin I was 0.07. Brain natriuretic peptide (BNP) was 147.03 pg/ml. Echocardiography on day 1 was normal (shortening fraction: 35%; ejection fraction: 66%). Diameters of the left and right coronary arteries were 0.24 and 0.20 cm (*Z* score, 2.0). Hence, she was suspected of having KD, and on day 2 of admission, before we could treat her with IVIG, she showed signs of shock, including increase in heart speed, cool extremities, oliguria, tachypnea, and hypotension (70/33 mmHg) requiring mechanical ventilation. She was immediately transferred to the intensive care unit. Electrocardiography (ECG) showed sinus tachycardia with alternation of T wave on leads II, III, and avF (Figure 1a). Chest X-ray showed bilateral lung field exudation and cardiomegaly. Arterial blood gas showed a lactate of 4.9 mmol/L. The urine output of the patient was < 0.5 ml/kg/h. She urgently received continuous renal replacement therapy (CRRT) in CVVHDF mode and therapy for septic shock. Echocardiography showed a depression of systolic function (EF 35%) with dilation of left ventricular end diastolic dimension (LVDd 3.7 cm) and severe tricuspid valve regurgitation (TR; Figures 1b,c). Cardiac index (CI) was 1.7 L/min/m^2^. Despite 0.6 μg/kg/min of both epinephrine and norepinephrine, her blood pressure couldn't be maintained (range, 57–69/31–40 mmHg). BNP was >15,000.00 pg/ml, and troponin I was 0.55. Laboratory findings and clinical features concluded the diagnosis of cardiogenic shock resulting from KDSS. Four hours later, she was placed onto central VA ECMO via neck cannulation.

**Figure 2:**
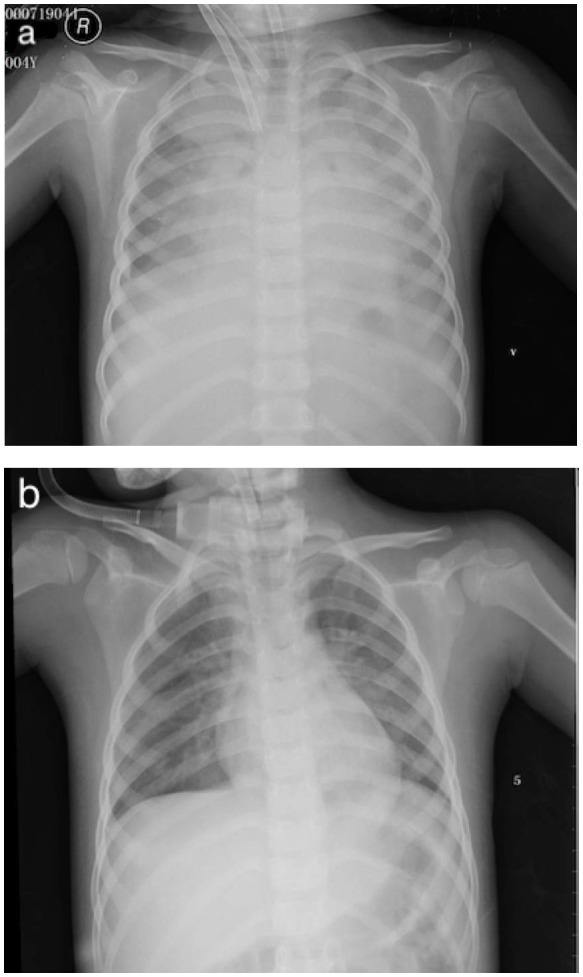
**(a)** V-A ECMO via neck cannulation with a 15-Fr cannula in the right atrium and a 12-Fr cannula in the right common carotid aorta. **(b)** Chest X-ray post-ECMO.

A 15-Fr cannula (Medtronic or Edward's Lifesciences, Irvine, CA, USA) was placed in the right atrium and a 12-Fr cannula (Medtronic or Edward's Lifesciences, Irvine, CA, USA) was placed in the right common carotid aorta (Figure 2a). The fraction of inspiration O_2_ (FiO_2_) was 1.0, blood flow was 0.8 L/min, and gas sweep flow was 1.0 L/min. Treatment with 2 days of IVIG (1 g/kg per day) and 5 days of intravenous methylprednisolone (2 mg/kg per day) were initiated right away. A mean blood pressure level of 50–60 mmHg was maintained by the initial flows of ECMO, and the serum lactate was normalized within 8 h. After 2 days of IVIG, her body temperature still fluctuated, and she was considered to be IVIG-resistant; she received plasma exchange (PE) for 6 h to reduce the inflammatory and immune reaction. Aspirin was maintained for 3 days at a dose of 30 mg/kg, and then at a dose of 5 mg/kg since. Fever settled on day 6.

The cardiac function of the patient recovered promptly on ECMO, and blood flow was reduced to 0.18 L/min and gas sweep flow was 0.3 L/min after 76 h, which meets the standard of separation of ECMO. Figure 2b showed the X-ray post-ECMO. The patient's vital signs tended to be stable during ECMO, with proper blood pressure, adequate urine output, and resolution of heart failure (EF 46% at day 2 ECMO and EF 54% at day 3 ECMO). Bilateral blood culture detected no infections spreading through the bloodstream. Table 1 shows laboratory findings pre- and post-ECMO. On day 9, the mechanical ventilation was separated and she was discharged on day 22. Follow-up within 3 months demonstrated that the cardiac and vascular functions were in the normal range (EF 65%, coronary arteries; *Z* score, 2.0).

**Table 1:** Laboratory findings pre- and post-ECMO.

**Day of admission**	**1**	2^*^	**3**	**4**	**5^#^**	**6**
WBC ( × 10^9^/L)	12.50	17.05	30.75	13.91	23.51	22.80
*N* (%)	70.8	81.2	82.5	84.2	89.6	77.8
PLT ( × 10^9^/L)	121	118	210	112	112	116
AST (mmol/L)	54	50	74	591	2,720	633
ALT (mmol/L)	38	52	50	251	1,060	640
CRP (mg/L)	127	>180	>170	117	107	47
Albumin (g/L)	38.17	27.12	31.76	39.13	38.31	42.53
Sodium (mmol/L)	129	129	132	135	139	141
BNP (pg/ml)	147.03	>15,000.00	>15,000.00	>15,000.00	11,446.35	7,723.41
Troponin I	0.07	0.55	1.08	0.57	0.33	0.15

* *ECMO was initiated*.

# *ECMO was separated*.

## Discussion

KD is usually regarded as a type of systematic inflammatory vasculitis, of which coronary artery lesions (CALs) are the most common cardiovascular complications (2). Occasionally, life-threatening cardiac complications may occur during the acute phase of KD or even later as a consequence of myocardial involvement (9). KD is diagnosed according to the American Heart Association (AHA) guidelines (11). Acute cardiac dysfunction was observed at nearly 20% of KD cases, which is considered to be related to the higher incidence of coronary artery dilatation (9).

KDSS is a manifestation of KD that is uncommonly seen, defined as systolic hypotension or signs of poor perfusion (12). Capillary leakage caused by vasculitis and cytokine dysregulation due to inflammatory syndrome may be responsible for KDSS, although the actual cause is still unclear (13). Recently, one case of a KD patient was reported with suspected systemic capillary leak syndrome (SCLS) (3). As in our case, the patient could have experienced plasma extravasation due to such capillary leakage, and myocardial destruction may be linked to the elevated BNP and troponin I level. In a recent study, researchers suggested that acute LV dysfunction and mitral regurgitation (MR) are associated with inflammation-related laboratory findings, such as decreased ALB and elevated ESR (14). Cardiac abnormalities without coronary artery involvement and CALs in acute KD may develop from a common pathological mechanism relating to systemic inflammation (14). Qiu et al. (2) spoke highly of the specificity of cardiac injury markers compared to inflammatory indicators in KD patients complicated with cardiac dysfunction and thus concluded that the blood pressure of such patients who show signs of accelerated heart rate, diminished urine output, and cool extremities should be closely monitored and that more attention should be paid to their echocardiography and laboratory findings. Among patients with KDSS, the lower serum albumin, sodium, and potassium concentrations may be related to protein leakage caused by vascular inflammation (2). In addition, Schuster et al. (15) suggested that the presence of a low level of serum sodium is associated with the presence of shock. Further studies are necessary to assess whether the presence of moderate/severe hyponatremia may be used to predict KDSS. KDSS is often associated with more severe laboratory markers of inflammation and higher risk of coronary arterial dilation (11). In recent studies, the profound inflammation may be associated with a higher incidence of CALs in KDSS patients compared with KD patients without KDSS (4), while there were opposite results in other series (16). In our case, there were no positive coronary artery findings through the whole stage of her hospitalization, even during follow-up. Further investigation of the mechanism of KDSS cardiac dysfunction might be helpful for us to understand the consequences of KD in the long run (14).

Manifestation of KDSS can be similar with clinical features of toxic shock syndrome, when hypotension was most commonly observed (17). The profound cardiac failure led to shock, which was difficult for us to distinguish from toxic septic shock at the beginning. As in our case, the profound shock manifested on day four of the illness, before the patient could meet the diagnostic criteria of complete KD. The high degree of the inflammatory indexes led us to the diagnosis of toxic septic shock. The echocardiography finding in toxic septic shock patients at early stage is mostly hyperdynamic systolic function of LV without myocardial dysfunction (18). This finding in toxic septic shock may suggest a compensating reaction to physiological shock, rather than a direct myocardial involvement (18). As a result, emergency bedside echocardiography and Uscom were used to detect ejection fraction and CI, which were helpful for us to distinguish cardiogenic shock caused by KDSS from septic shock.

Given the diagnosis of the shock in our case, we used V-A ECMO via neck cannulation. We searched through the Extracorporeal Life Support Organization (ELSO) Registry database. ECMO applications for 23 KD patients were reported from 1999 to 2017. Among those, 9 were supported for respiratory indication, while the other 14 underwent ECMO support for indications of cardiac or ECMO CPR (ECPR) (9, 19). In the accessible literature, we found that VA ECMO cardiac or ECPR support for KD cases was scarcely reported. Although ELSO is the authority in terms of ECMO use worldwide, it may be incomplete. There may still be other cases of VA ECMO support for KD that were not reported. Table 2 compares the mean clinical features of patients from the ELSO database and our case, which shows that the ECMO flows we used were similar to that used in such population.

**Table 2:** Clinical features in ELSO data comparing our case.

**ELSO Data (Cardiac/ECPR) for Kawasaki disease: 1999–2017**			**Our reported case**
	**Median**	**Range**	
Age (days)	248	51–4,625	1,645
Weight (kg)	8	5–50	18
Ventilation time prior to ECMO (h)	22	2–201	5
Blood flow 4 h (ml/kg/min)	85.5	46–156	50
Blood flow 24 h (ml/kg/min)	95	39–155	55

Taddio et al. (4) reported that a reduced ejection fraction was frequently seen in KDSS patients. Most cardiovascular complications recovered rapidly during the sub-acute or convalescent phase, and no patient presented persistent CALs. Some researchers have concluded that the prompt LV function resolution was associated with the modulation of immune-mediated processes (14). As in our case, we detected such cardiac abnormalities without CALs, including acute LV systolic dysfunction and severe TR, which were transient under VA ECMO support. The situation in our case was similar to prior studies (14). At the acute stage of KDSS, it may be misdiagnosed as toxic septic shock and be treated with inappropriate fluid resuscitation, hence worsening cardiac function or delaying IVIG treatment. As in our case, the cardiogenic shock occurred on day 4 of fever, before the patient met the diagnostic criteria of complete KD and also earlier than we usually initiate IVIG treatment as we deal with KD patients without KDSS. According to the AHA guidelines, such patients should be treated with high-dose IVIG (2 g/kg given as a single intravenous infusion) within 10 days of illness onset but as soon as possible after diagnosis (11). As in our case, the IVIG was not given in one large dose, which may be responsible for the rapid deterioration of the patients' situation. To sum up, it is vital to increase the knowledge of KDSS early recognition. Further studies on the correct use of IVIG for KDSS might help reveal the approach of preventing such patients from having irreversible outcomes.

## Concluding Remarks

KDSS can cause life-threatening cardiac dysfunction, potentially complicated by coronary artery involvement. Clinicians should pay closer attention to KDSS, and IVIG should be initiated as a single intravenous infusion as soon as possible in such patients. Emergency bedside echocardiography and Uscom may serve as sensitive methods for early differentiation between KDSS and toxic septic shock. Although rarely reported, VA ECMO is useful as a lifesaving procedure for cardiac support in such cases.

## Data Availability

All datasets generated for this study are included in the manuscript and/or the supplementary files.

## Ethics Statement

This study was carried out in accordance with the recommendations of International Ethical Guidelines for Health-related Research Involving Humans, Council for International Organizations of Medical Sciences (CIOMS) with written informed consent from all subjects. All subjects gave written informed consent in accordance with the Declaration of Helsinki. The protocol was approved by the Institutional Review Board of Shanghai Children's Hospital.

## Author Contributions

HZ and LX prepared the entire manuscript. TX contributed to the conception of the case and revised the manuscript. HZ organized the database. All authors are responsible for manuscript revision and approved the submission of the case.

### Conflict of Interest Statement

The authors declare that the research was conducted in the absence of any commercial or financial relationships that could be construed as a potential conflict of interest.
